# A review of current evidence for mild behavioral impairment as an early potential novel marker of Alzheimer’s disease

**DOI:** 10.3389/fpsyt.2023.1099333

**Published:** 2023-04-27

**Authors:** Piaopiao Jin, Jiaxi Xu, Zhengluan Liao, Yuhan Zhang, Ye Wang, Wangdi Sun, Enyan Yu

**Affiliations:** ^1^The Second School of Clinical Medicine, Zhejiang Chinese Medical University, Hangzhou, China; ^2^Department of Geriatric VIP No. 3 (Department of Clinical Psychology), Rehabilitation Medicine Center, Zhejiang Provincial People’s Hospital, Affiliated People’s Hospital, Hangzhou Medical College, Hangzhou, China; ^3^Department of Psychiatry, Zhejiang Hospital, Hangzhou, China; ^4^Department of Psychiatry, The Cancer Hospital of the University of Chinese Academy of Sciences (Zhejiang Cancer Hospital), Institute of Basic Medicine and Cancer (IBMC), Chinese Academy of Sciences, Hangzhou, China

**Keywords:** mild behavioral impairment, Alzheimer’s disease, mild behavioral impairment checklist, risk factor, type of review, prodromal

## Abstract

Mild behavioral impairment (MBI) is a neurobehavioral syndrome that occurs in the absence of cognitive impairment later in life (≥50 years of age). MBI is widespread in the pre-dementia stage and is closely associated with the progression of cognitive impairment, reflecting the neurobehavioral axis of pre-dementia risk states and complementing the traditional neurocognitive axis. Despite being the most common type of dementia, Alzheimer’s disease (AD) does not yet have an effective treatment; therefore, early recognition and intervention are crucial. The Mild Behavioral Impairment Checklist is an effective tool for identifying MBI cases and helps identify people at risk of developing dementia. However, because the concept of MBI is still quite new, the overall understanding of it is relatively insufficient, especially in AD. Therefore, this review examines the current evidence from cognitive function, neuroimaging, and neuropathology that suggests the potential use of MBI as a risk indicator in preclinical AD.

## 1. Introduction

There are currently more than 55 million people living with dementia worldwide, with an average of one new dementia patient every 3 s (1, 2). By 2050, the number of people affected is expected to reach 139 million. Alzheimer’s disease (AD) is an irreversible neurodegenerative disorder characterized by progressive cognitive dysfunction and behavioral impairment (3). AD is the most common cause of dementia estimated to account for 60–80% of dementia cases (2). AD poses a serious threat to the health and safety of older adults and places a heavy burden on families, society, and healthcare systems (2). No drugs can effectively delay AD (4), so early diagnosis and intervention are particularly important.

Neuropsychiatric symptoms (NPS) are almost universal during the dementia process (5–8), symptom severity may fluctuate, typically becoming more severe with advancing disease pathology (8–11), increasing the risk of death in patients with dementia and caregiver burden for those providing care (12, 13). Studies have found that neuropsychiatric symptoms occur in the pre-dementia period, even before the onset of cognitive symptoms (14–16); not only in frontotemporal dementia (FTD) (17, 18), but also in AD (14, 17, 19).

In recent years, research is increasingly focusing on early recognition and intervention of NPS in AD. So far, multiple longitudinal and cross-sectional studies have supported an association between NPS and AD progression. In a cross-sectional study (20), Belgian researchers identified three similar behavioral syndromes (a depression, a psychosis, and an agitation syndrome) in patients with mild cognitive impairment (MCI) and AD. In an analysis of the behavioral symptoms of MCI, Stefan et al. found that the general severity of MCI behavioral symptoms ranged between normal aging and AD patients, with aggression, affective disorders, and anxiety being the most common (21). In a large MCI cohort, 24.9% of 1,821 MCI patients progressed to AD after a mean follow-up of 1.16 years, and the presence of any NPS and any depressive symptoms at baseline was observed to increase the risk of dementia and AD by 30–40% after a mean follow-up of 1.5 years (22). Interestingly, in another follow-up study of more than 5 years, it was found that NPS preceded a cognitive diagnosis for the majority of people who developed cognitive decline (MCI and dementia) (15). The study longitudinally analyzed NPS in 1,998 subjects who progressed from cognitively normal (CN) to MCI or dementia [as assessed using the Neuropsychiatric Inventory (NPI)] and found that among those who eventually developed AD, 30% developed NPS before MCI, whereas 42% developed NPS after MCI but before dementia. In addition, the prevalence of NPS in the year prior to the MCI or dementia diagnosis was 45–60%. Despite the fact that the volunteers in this study had a high family history of dementia, it is clear that the presence of new-onset NPS in cognitively normal older adults is likely to mean that they are at an increased risk of developing cognitive impairment.

The past decade has seen mild behavioral impairment (MBI) as a potential preclinical manifestation of neuropathology that reflects early behavioral signals of dementia (14). MBI refers to later-life emergent psychiatric and behavioral symptoms in the absence of typical clinical cognitive symptoms in dementia (23). MBI is a relatively new concept, first proposed by Taragano and colleagues (19, 23). In addition, leveraging the well-known behavioral prodrome of FTD (17, 18), Taragano et al. first proposed that MBI has a higher risk of dementia conversion than MCI, especially in FTD. MBI (specifically in those without cognitive symptoms) may be the transitional state between normal aging and dementia and may help in early prevention and targeted treatment of dementia (14).

Although research on MBI started later in the context of AD than in FTD, the role of MBI in the prodromal period of AD has received increasing attention. Importantly, MBI has been included in phase 2 of the National Institute on Aging and Alzheimer’s Association (NIA-AA) AD research framework, along with early cognitive signals, that is, subjective cognitive decline (SCD) (24). In this review, we will describe the progress of MBI research in AD and show its potential as a marker for preclinical AD based on current evidence.

## 2. The ISTART-AA MBI diagnostic criteria

**Figure 1:**
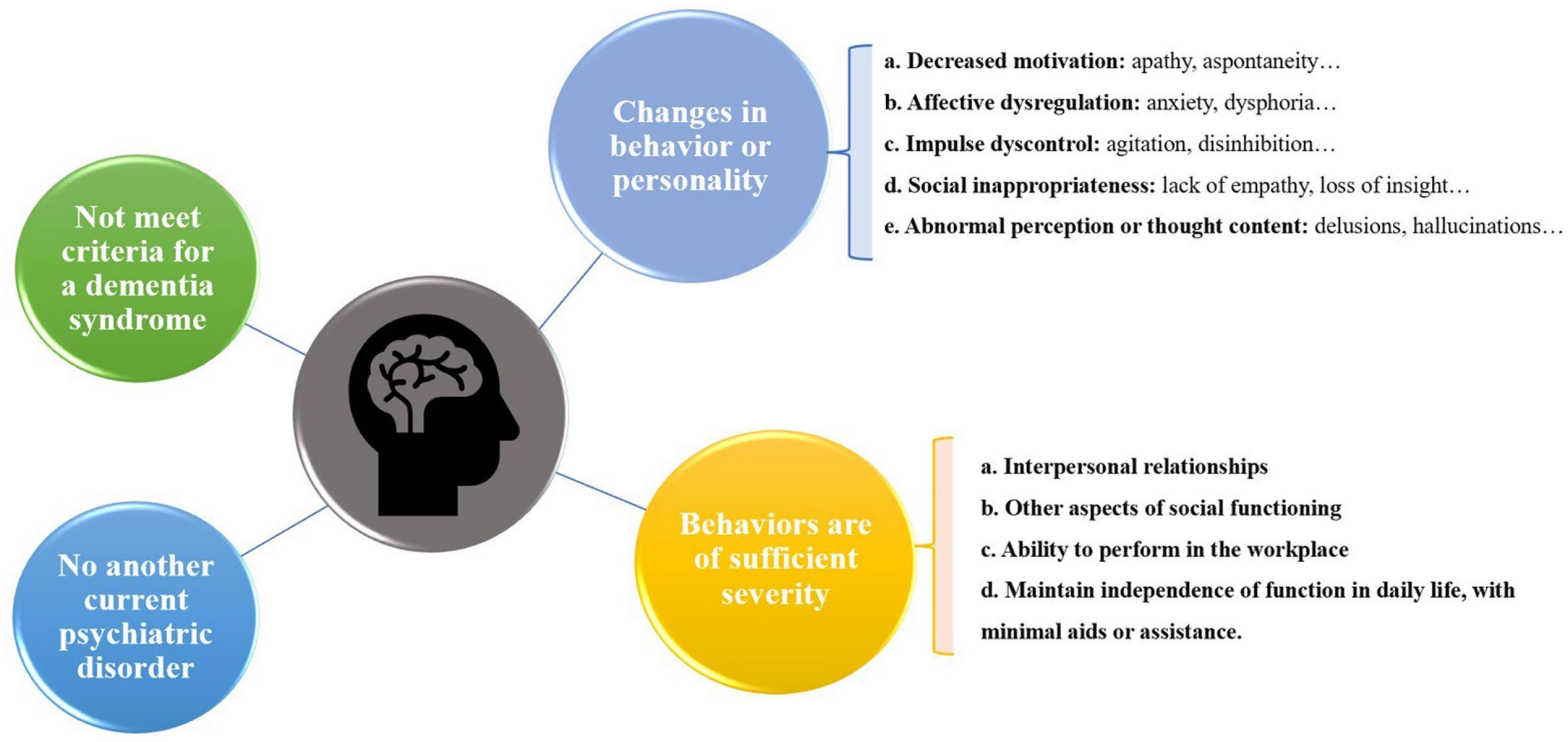
Diagram of the ISTART-AA MBI diagnostic criteria.

Based on the need for early identification and treatment of neurodegenerative disorders, the International Society to Advance Alzheimer’s Research and Treatment (ISTAART) NPS Professional Interest Area has expanded the Taragano MBI diagnostic criteria and proposed new diagnostic criteria for research, the ISTART-AA MBI diagnostic criteria (25) (Figure 1).

The ISTAART-AA criteria for the diagnosis of MBI require the observation of behavioral or personality changes that start later in life (age ≥ 50 years). These changes are inconsistent with the person’s usual behavior or personality, persist at least intermittently for ≥6 months, and can be demonstrated by changes in at least one of MBI domains of decreased motivation, affective dysregulation, impulsive dyscontrol, social inappropriateness, and abnormal perception or thought content. Notably, the criteria emphasize that an individual with MBI achieves at least minimal functional impairment in social, occupational, or interpersonal functioning and that the impairment must be attributable to NPS rather than cognitive decline (25). In addition, MBI is only diagnosed in people without dementia but can be co-diagnosed with MCI (25).

As described in the ISTART-AA criteria, MBI is independent of the traditional neurocognitive axis (i.e., MCI) and represents the neurobehavioral axis of neurodegenerative disorders (25). MBI and MCI are not in competition and are not either-or but exist in parallel and can occur simultaneously or separately. The new criteria clearly state that MBI is not limited to FTD alone but is a precursor to all dementias, including AD (25, 26). In addition, the description of MBI-specific behavioral domains may help to distinguish behavioral endophenotypes, thus potentially improving predictive accuracy and potentially influencing prognosis.

## 3. Epidemiology and measurement

MBI is seen as a late-life transition state between normal aging and the prodromal stage of dementia and increases the risk of dementia in cognitively normal individuals or individuals with MCI (23, 25).

In a community-based study, Mortby et al. used NPI to assess NPS in 1,417 older adults (aged 72–79 years) across the cognitive functioning spectrum, including subjects at all stages of CN, cognitively normal but at risk (CN-AR), MCI, and dementia (10). The study reported that NPS is prevalent over the entire cognitive spectrum (30.8–80%) and is associated with a 3-fold, 2-fold and 1.5-fold increased risk of dementia, MCI and CN-AR, respectively (10). Although this study demonstrated a high prevalence of NPS and its association with the risk of cognitive impairment, it lacked longitudinal data and was not conducted using the MBI diagnostic framework. Following the publication of the ISTAART-AA diagnostic criteria for MBI, Mortby et al. conducted the first epidemiological investigation based on these criteria (27). They developed the MBI conversion matrix by mapping 10 of 12 NPI items (delusions, hallucinations, agitation/aggression, depression/dysphoria, anxiety, elation/euphoria, apathy/indifference, disinhibition, irritability/lability, aberrant motor behaviors) onto the MBI domains to estimate MBI frequency (27). Mortby et al. estimated the prevalence of MBI across the cognitive spectrum from CN, CN-AR, to MCI to be 27.6, 43.1, and 48.9%, respectively, with an overall prevalence of 34.1% (27). The construction of an MBI diagnostic framework can help in the early identification of individuals at an increased risk of dementia without significant cognitive symptoms.

In fact, several studies using the MBI conversion matrix developed by Mortby et al. have shown that MBI appears to be more prevalent in clinical samples than in community samples. Studies have shown that 19.6% of patients with SCD and 50% of patients with MCI in psychiatric outpatient clinics meet the diagnostic criteria for MBI (28). In memory clinics, the prevalence of MBI in patients with SCD and MCI can be as high as 76.5 and 85.3%, respectively (29). Moreover, caregiver burden has been shown to be 3.35 times higher when MBI is present (29). Nevertheless, NPI used in the above study only covers the preceding 1 month, which is shorter than the 6-month observation period required for the diagnosis of MBI and does not focus on the persistence of symptoms, thus possibly overestimating the prevalence of MBI (28, 29). In another epidemiological survey of a memory clinic sample (30), the researchers extended the reference time of NPI to 6 months and used four diagnostic criteria for MBI to improve the accuracy (30). In this study, MBI prevalence of approximately 41.13% was observed in individuals with amnestic MCI (aMCI), and the odds of having conditions such as hypertension and diabetes were higher in SCD or MCI patients with MBI. In addition, in this clinical sample, patients with MBI had higher odds of having multimorbidity, compared to patients without MBI (30). Although actively extending the observation time of the scale may improve the accuracy of the diagnosis of MBI (30), it still differs from the official MBI diagnostic criteria because the NPI is mainly used in patients with dementia and may not be applicable in patients with pre-dementia (31–33).

Specific scales are needed to meet the needs of research in the field of MBI. The Mild Behavioral Impairment Checklist (MBI-C) was developed based on the ISTART-AA MBI criteria, tailored for people with pre-dementia, to better describe and measure the MBI (34). The MBI-C is divided into 5 domains: decreased motivation, affective dysregulation, impulse dyscontrol, social inappropriateness, and abnormal perception/thought, corresponding to the MBI domains (34). The MBI-C is a simple scale consisting of 34 questions, each of which requires a “yes” or “no” answer to determine the presence or absence of symptoms, and if “yes,” then a rating of the severity of the symptoms on a scale of “1-mild, 2-moderate, or 3-severe” is completed, with the severity scores summed to give a total score ranging from 0 to 102 (34). Note that each symptom is answered “yes” only if it has been present intermittently or consistently for at least 6 months later in life (≥50 years) and is different from its previous long-term behavioral pattern (34). The MBI-C was developed to measure the prevalence of MBI as well as to assess the risk of cognitive decline and dementia based on overall and domain scores, primarily completed by informants such as family members and spouses (34). The MBI-C has been translated from the original English version into more than a dozen languages and has been used in research (35–42) (The MBI-C is available for free at http://www.MBItest.org).

Several studies have recently begun to use the MBI-C to estimate the prevalence of MBI, and because the MBI-C is more stringent about symptoms, studies have observed more conservative and accurate results. Mallo et al. (36, 37) obtained MBI-C scores from primary care patients by telephone interviews, and their results indicated a prevalence of 14.2 and 5.8% for MBI in MCI and SCD, respectively. The prevalence of MBI in elderly people with normal cognition is 10% (11). In addition, similar to the results previously observed using the NPI, a higher prevalence of MBI was observed in the clinical sample using the MBI-C scale (30, 42–44), with more than half of MCI patients suffering from MBI (42, 44).

Notably, there were also differences in the prevalence of MBI domains. Affective dysregulation and impulse dyscontrol have been shown to be the two most common individual MBI domains in multiple studies (27–30, 36, 37, 39, 42–44), independent of the scale used. A recent meta-analysis (45) pooled 10 studies conducted among 9,758 CN, 1,057 SCD, and 1,252 MCI subjects, totaling 12,067 subjects, to estimate the prevalence of each domain. The results showed that the prevalence of the two most common MBI domains, affective dysregulation and impulse dyscontrol, was 32.84% (95% confidence interval [CI] 24.44–42.5%) and 26.67% (95% CI 18.24–37.23%), respectively, followed by decreased motivation and social inappropriateness at 12.58% (95% CI 6.93–21.75%) and 6.05% (95% CI 3.44–10.42%), respectively, while abnormal perception/thought had the lowest prevalence at 2.81% (95% CI 1.67–4.69%). There are also sex differences between the domains of MBI (28, 46, 47), with decreased motivation and impulse dyscontrol more common in males, and affective dysregulation and abnormal perception/thought more common in females. In addition, the severity of MBI increases with age (48) and is associated with frailty in older adults (39). Overall, the worse the cognitive status, the higher the prevalence (30, 36, 37, 42–44) and severity (38, 39, 43, 46, 49–51) of MBI. This suggests that the presence of the MBI and its domains may predict different levels of dementia risk.

However, the cut-off points for the diagnosis of MBI for the total score and each domain score of the scale are still in the research stage. Regarding the total score of the MBI-C, setting cut-off points of 6.5 in MCI (36) and 8.5 in SCD (37) for the diagnosis of MBI has demonstrated good sensitivity and specificity and these thresholds have been used in several studies (11, 16, 39, 42, 43). Different optimal cut-off points have been reported in other studies. For a cognitively normal community sample, one study set the MBI-C cut-off at 6 and suggested that its optimal cut-off point for identifying cases would be between 2 and 8 (48). Furthermore, both self-reported and informant-reported MBI-Cs have shown validity and feasibility (11, 36, 37, 49) and can even be used for remote online assessment (49, 52). However, no similar studies have been conducted on individual MBI domain scores. The use of MBI-C in clinical and research settings has been important for the identification of MBIs. Future studies in different populations are needed to determine the cut-off points for the total scale scores and especially for the individual MBI domain scores. Prospective studies on the relationship between MBI identified by the MBI-C and dementia risk are warranted to help find the timing for early identification and intervention.

In addition, in AD, it has been suggested that the MBI-C can be used as a screening tool for AD dementia or even to differentiate the severity of dementia, and the optimal cut-off point for identifying AD is 6/7 (38). We know that MBI is defined according to the criteria as the presence of behavioral changes in at most people with MCI (25). What’s more, the MBI Checklist is a case ascertainment tool for MBI and not AD dementia. While MBI is considered a risk factor for transition to AD dementia, the MBI Checklist does not itself screen for AD dementia or differentiate the severity of dementia. It is therefore not logical that it would be used in participants with AD (rather the use of a tool such as the NPI in AD is more appropriate).

## 4. MBI and cognitive function correlation

To better assess the role of MBI and its domains in preclinical AD, existing NPI data can be used. A retrospective study of psychiatric outpatients using the NPI for the diagnosis of MBI found that the hazard ratio (HR) for dementia was higher for MBI than other psychiatric diseases (HR = 8.07, 95% CI: 4.34–15.03, *p* < 0.001), and those with affective disorders had an increased risk of developing dementia among MCI patients (HR = 1.646) (28). This result is interesting in that we can see that MBI predicts a higher risk of dementia, and the predictive power seems to be different for different MBI domains, which informs the subsequent predictive power of dementia risk for individual MBI domains. Of course, the study has limitations, such as the follow-up time of some subjects was insufficient (minimum 1 month) and the retrospective study design may have some insufficient information.

Using data from the National Alzheimer’s Coordinating Center (NACC) database, participants were divided into four groups (MBI-SCD-, MBI-SCD+, MBI + SCD-, or MBI + SCD+) to observe the degree of clinical dementia rating (CDR) decline after 3 years and found that MBI was associated with poorer executive function, attention, and episodic memory regardless of cognitive status (53). Patients with both MBI and MCI were found to have a greater risk of dementia compared to patients with MBI or MCI alone (53). The authors converted the NPI score to MBI-C domains [using the MBI conversion matrix developed by Mortby et al. (27)] and ensured symptom persistence for two consecutive measurements, but they risked overestimating MBI by treating all patients with non-zero scores as MBI cases. Undeniably, the authors demonstrated that MBI can serve as a predictor of dementia risk as SCD, and that the two may be complementary constructs. In addition, it seems necessary to further develop and refine the operationalized matrix in order to improve the accuracy of the previous existing retrospective evaluation based on population and clinical studies.

In another study using the MBI-C as a measurement tool, the investigators divided the subjects into three groups based on MBI-C scores: NS [No Symptoms group, MBI-C = 0], NPS [Intermediate NPS group; MBI-C = 1–8], and MBI [MBI group >8] (11). This study found that cognitively normal subjects with MBI showed faster decline in attention and working memory; in particular, declines in working memory may be particularly associated with preclinical AD (11). The discovery of these “unique cognitive phenotypes” is of great interest and points the way to follow-up studies. Continuation of this study is necessary, especially to improve biomarker detection and long-term follow-up to confirm the risk of MBI-related cognitive decline transforming into MCI or dementia. This may help allow time for clinical intervention. A prospective cohort study of community-dwelling older adults observed that nonpsychotic symptoms strongly predicted MCI events after 5 years of follow-up, with the strongest predictor being agitation/aggression (hazard ratio [HR] = 3.06, 95% CI = 1.89–4.93), followed by apathy (HR = 2.26, 95% CI = 1.49–3.41), whereas delusions and hallucinations did not predict the occurrence of MCI (54). Similar to previous observations (28), the domains of the MBI correlated with the progression of cognitive impairment to varying degrees. It is more advantageous to use ISTAART-AA MBI diagnostic criteria and MBI-C in MBI research because they are designed for this purpose and can generate domain scores for relevant analysis.

More critically, a recent large study stratified cognitively normal participants from the National Alzheimer’s Coordinating Center (NACC, *N* = 11,372) by MBI status using the NPI and reported that MBI was a significant predictor of progression to clinically diagnosed AD (HR = 1.75) and neuropathologically confirmed AD (HR = 1.59) (55). The MBI domain was also associated with clinically diagnosed AD, with the greatest effect on psychotic symptoms (HR = 6.49) (55). This evidence further supports the hypothesis that early MBI can predict the progression of AD independently of cognitive symptoms.

## 5. MBI and AD genetic correlation

Clinical evidence supports the use of MBI and its domains as non-cognitive markers of preclinical diseases. Emerging evidence has found a certain correlation between MBI and AD in pathophysiological mechanisms. Andrews et al. constructed genetic risk scores (GRS) from 25 AD risk loci and found that five loading risk loci (APOE, MS4A, BIN1, EPHA1, NME8, and ZCWPW1) were associated with MBI domains. Research suggests a shared genetic etiology between MBI and cognitive problems that has traditionally been observed in AD (56). Nathan et al. stratified more than 5,000 older adults with SCD according to MBI status (MBI + or MBI -) and found more ApoE4 homozygotes in the MBI + group compared to the MBI – group (57). In addition, a sample of 4,458 individuals aged over 50 years without dementia was stratified by MBI status and found that genetic risk for AD was observed to be correlated with cognitive performance in individuals with MBI, but this association was not observed in individuals without MBI (48). These findings are highly encouraging and contribute to our understanding of the underlying pathophysiological features of MBI. Nevertheless, these studies have not been able to draw firm conclusions about the etiology of genetic and cognitive associations, and further research is required.

## 6. MBI and AD imaging features correlation

By measuring volumes of several parts of frontal lobes in patients with MCI (regardless of etiology), MCI due to AD, AD dementia, and behavioral variant frontotemporal dementia (bvFTD), Cajanus et al. found that smaller volumes in the subcallosal area were associated with higher disinhibition and aberrant motor behavior scores, as well as the total behavioral symptoms score across the diagnostic groups (58). They concluded that damage to the subcallosal area may be a common neuroanatomical area for behavioral symptoms in neurodegenerative diseases independent of the specific type of dementia (58). In another study, the authors aimed to find out MRI correlates of impulse dyscontrol in normal controls, MCI, and AD patients (59). T1-weighted and diffusion-tensor magnetic resonance imaging (DTI) data from individuals with and without impulse dyscontrol were compared. Impulse dyscontrol was associated with: (1) lower fractional anisotropy (FA), and greater mean, axial, and radial diffusivity in the fornix; (2) lesser FA and greater radial diffusivity in the superior fronto-occipital fasciculus; (3) greater axial diffusivity in the cingulum; (4) greater axial and radial diffusivity in the uncinate fasciculus; and (5) gray matter atrophy, specifically, lower cortical thickness in the parahippocampal gyrus (59). The frontal striatal network appears to play a key role in mediating these behaviors (60). Recently, a study found that the total MBI-C and affective dysregulation domain scores were negatively correlated with functional connectivity of the left posterior parietal cortex with the right middle frontal gyrus (41). Moreover, in a clinical cohort of non-demented older adults, MBI, and especially the impulse dyscontrol and decreased motivation domains, was associated with atrophy in two medial temporal lobe regions, that is, the entorhinal cortex and hippocampus (61), suggesting that early MBI involves temporal but not frontal regions. Another study confirmed that structural changes in the gray matter in MBI patients occurred mainly in the left temporal lobe (62), supporting the association of the temporal lobe with NPS. The difference in the results may be explained by the “agitation circuit” proposed by Rosenberg et al. (63), which consists of the frontal cortex, anterior cingulate cortex, orbitofrontal cortex, amygdala, hippocampus, and insula (63). The region of damage described above resembles the established pattern of atrophy in AD (64, 65), indicating that MBI is closely related to early AD neurodegeneration and could be an early manifestation of neurodegeneration. Further sample size expansion and longitudinal studies are required to test this relationship (Table 1).

**Table 1:** Neuroimaging and neuropathology studies of MBI and AD.

References	Samples	Methods	measurement	Main findings	Conclusion
Cajanus et al. (58)	MCI, *n* = 58; AD, *n* = 45; bvFTD, *n* = 18	3T MRI	NPI	Negative correlation between some behavioral symptoms and the volume of the subcallosal area.	The subcallosal area may be common neuroanatomical area for behavioral symptoms.
Johansson et al. (74)	CSF Aß+: CN, *n* = 50; MCI, *n* = 53; AD, *n* = 62	CSF P-tau181; [18 F]RO948 tau-PET	MBI-C	Tau deposition in the entorhinal cortex was associated with MBI-C scores. In Aß+ CU cases, entorhinal tau deposition was predicted by MBI-C. In Aß+ MCI subjects, ADAS-DR predicted the level of entorhinal tau deposition.	MBI is one of the earliest clinical symptoms associated with early tau pathology during the preclinical stages of AD.
Lussier et al. (69)	CN, n=96	3T MRI; [18 F]AZD4694 Aβ-PET; [18 F]MK6240 tau-PET	MBI-C	Mild behavioral impairment is associated with β-amyloid but not tau or neurodegeneration in cognitively intact elderly individuals.	MBI, measured by the MBI-C, constitutes an early clinical manifestation of AD pathophysiology, before cognitive decline is detected.
Gill et al. (59)	with impulse dyscontrol, *n* = 80; without impulse dyscontrol, *n* = 123	MRI from ADNI database	transform NPI into MBI domains	Impulse dyscontrol was associated with: lower FA, and greater mean, axial, and radial diffusivity in the fornix; lesser FA and greater radial diffusivity in the superior fronto-occipital fasciculus; greater axial diffusivity in the cingulum; greater axial and radial diffusivity in the uncinate fasciculus; gray matter atrophy, specifically, lower cortical thickness in the parahippocampal gyrus.	Supporting that impulse dyscontrol symptoms as an early manifestation of AD.
Matsuoka et al. (41)	CN, *n* = 30; amnestic MCI, *n* = 13	3T MRI	MBI-C	Negative correlation between the MBI-C total score and affective dysregulation domain score and FC of the left posterior parietal cortex with the right middle frontal gyrus.	FC network dysfunction may be associated with cognitive impairment in MBI and conversion of MBI to dementia.
Shu et al. (62)	MBI, *n* = 16; healthy controls, *n* = 18	3T MRI	MBI-C	Atrophy in the left frontal cortex and right thalamus in MBI patients is in line with frontal-subcortical circuit deficits.	MBI might be an early harbinger for subsequent cognitive decline and dementia.
Matuskova et al. (61)	SCD, *n* = 37; MCI, *n* = 79	1.5T MRI	MBI-C	ERC was associated with MBI-C total score and with impulse dyscontrol score. HV was associated with decreased motivation and impulse dyscontrol score.	The MBI-C may potentially help further identify individuals at-risk of developing AD dementia.
Miao et al. (72)	CN, *n* = 86; MCI, *n* = 53	plasma Aβ	MBI-C	Lower plasma Aβ42/Aβ40 was associated with higher MBI total score and greater affective dysregulation, but not with impaired drive/motivation or impulse dyscontrol MBI domains.	Incorporating MBI into case detection may help capture preclinical and prodromal Alzheimer’s disease.

CN=cognitively normal; SCD=subjective cognitive decline; MCI=mild cognitive impairment; AD=Alzheimer’s disease; NPI=the Neuropsychiatric Inventory; MBI-C=the Mild Behavioral Impairment Checklist; MRI=Magnetic Resonance Imaging; NPS=neuropsychiatric syndrome; Aß+=ß-amyloid positive; CSF=Cerebrospinal Fluid; PET=positron emission tomography; ADAS-DR=the Alzheimer's Disease Assessment Scale - Cognitive subscale delayed word recall; FA= fractional anisotropy; FC= frontoparietal control; HV=hippocampal volume; ERC=entorhinal cortex; OFC=the orbitofrontal cortex.

## 7. MBI and AD neuropathology correlation

The characteristic pathological changes of AD (i.e., β-amyloid deposition and pathological tau protein) distinguish AD from other neurodegenerative diseases (69–71), and its biomarkers are proxies for AD neuropathologic changes (24). Emerging biomarker evidence has provided a more specific link between MBI and AD neuropathology. In a recent sample of CN older adults conducted within the A/T/N research framework (67), higher total MBI-C scores were confirmed to be associated with increased global and striatal amyloid pathology using β-amyloid positron emission tomography (PET), the gold standard biomarker for AD. More critically, the most strongly associated regions observed in this study, particularly the frontal neocortex and striatum, correspond to those known to exhibit amyloid changes in the first stages of graded amyloidosis in AD (72, 73). These findings provide a landmark demonstration of the link between MBI and early AD pathology in a cognitively intact elderly population. The correlation of MBI symptoms with plasma Aβ42/Aβ40 (68) and plasma neurofilament light chain (NfL) (74), potential plasma biomarkers of AD, appear to reflect these associations. Unlike the absence of association with tau PET in this sample, however, in another cross-sectional study of 50 Aβ-positive CN subjects (66), MBI scores (rather than episodic memory impairment) were reported to be independently associated with early tau pathology determined using cerebrospinal fluid P-tau181 or tau PET. This may be the result of differences in sample selection, since abnormal tau deposition has to essentially occur in addition to abnormal Aβ deposition (75, 76). Regardless, cognition in these samples should be normal, suggesting that the MBI is independent of cognitive symptoms and represents an early manifestation of the neuropathological changes in AD. Certainly, there is a need to expand the sample size and range for further validation in longitudinal cohorts (Table 1).

Machine learning (ML) is a branch of artificial intelligence and computer science which focuses on the use of data and algorithms to imitate the way that humans learn, gradually improving its accuracy. Through the use of statistical methods, algorithms are trained to make classifications or predictions, and to uncover key insights in data mining projects. It’s a promising tool for the specific prediction of the development of early cognitive impairment and dementia (77). Recently, Canadian research teams have attempted to use ML to make specific predictions for patients in the progression of cognitive impairment (78). They extracted data on neuropsychiatric symptoms (NPI converted to MBI domains) and neuroimaging data from the ADNI database for 102 CN and 239 MCI subjects, obtaining more than 200 potential features that might predict future diagnostic status. Ultimately, the best ML model was found to correctly classify participants as maintaining normal or developing cognitive impairment with 84.4% accuracy (ROC-AUC = 0.86) in a binary classification (CN vs. MCI/AD) requiring only two features: the total MBI score and left hippocampal volume (78). The total MBI scores, followed by impulse dyscontrol and affective dysregulation, were the most predictive of future diagnoses (78). It is not difficult to see that ML can predict the progress of AD by combining the data of future cognitive, spiritual, and behavioral dimensions, and MBI shows its importance at this level.

## 8. Conclusion/future directions

It has long been recognized that NPS are prevalent in dementia, widespread across the cognitive spectrum, and associated with poorer outcomes, including heavier caregiver burden (79), lower quality of life (80), higher rates of institutionalization (81), worsening dementia, and even death (82). In 1996, the International Psychogeriatric Association defined the presence of NPS in dementia as the “behavioral and psychological symptoms of dementia” (83), with evidence from a number of studies. Nevertheless, there is no unifying concept of NPS that appears in the pre-dementia stage or even in cognitively normal individuals, nor has there been an abundance of relevant studies. Until recently, the formal introduction of the MBI concept (23) and the establishment of the ISTART-AA MBI diagnostic criteria (25) have provided a clear framework within which to study NPS as an early marker of dementia risk.

MBI has shown its early identification and predictive role in neurodegenerative diseases in the currently available studies. There is growing evidence of a correlation between MBI and AD progression. First, several cross-sectional and longitudinal cognitive assessments suggest that MBI, especially affective dysregulation and impulse dyscontrol, are predictive of cognitive decline in CN, SCD, and MCI (11, 15, 20–22, 28, 53–55, 78). To further explore the pathophysiological mechanisms of MBI associated with AD progression, MBI has been shown to be correlated with genetic etiology (48, 56, 57), altered brain function (41, 58–62), and neuropathological alterations in AD (66–68, 72–76). Taken together, these evidences lead us to speculate that various etiologies such as genetic factors cause neuropathological alterations in AD, and these alterations gradually cause changes in brain function, which in turn exhibit various clinical manifestations, manifesting as MCI and MBI in early stages, and may gradually progress to dementia in later stages. Therefore, we considered that MBI has a certain potential as an early clinical marker of AD.

It is worth noting that while the results of these studies show great potential for the use of MBI as a non-cognitive marker, it has to be emphasized that these studies often have small sample sizes. Specifically, both the specific brain regions corresponding to MBI symptoms and the cumulative association with AD pathology require validation using longitudinal data. In addition, the scope of sample selection and the uniformity of diagnostic tools are limitations. The MBI-C needs to be validated in a broader population, especially for independent studies of individual domains. Previous research has demonstrated that affective dysregulation and impulse dyscontrol are the two most common individual MBI domains (27–30, 36, 37, 39, 42–44). For independent studies of individual domains, we first recommend these more common domains, as it is easier to obtain research subjects than less common domains, and the corresponding findings will benefit more people first.

We consider that the main value of MBI currently lies in clinical research, which can help identify potential AD early, screen for suitable study subjects, help explore the pathogenesis of AD, find therapeutic targets and effective timing of clinical intervention, etc. In addition, part of the value of MBI is to alert clinicians to the early identification and management of NPS. In addition, part of the value of MBI is to alert clinicians to the early recognition and management of NPS. We encourage the importance of MBI, but it is not necessary to be overly concerned. As demonstrated in epidemiological surveys, NPS can occur at all stages of cognitive impairment, but MBI mostly occurs after MCI and at stages prior to AD (14–16). Based on current research, we should be more concerned about those patients with co-occurring MBI and MCI who have the relatively highest probability of developing dementia than MBI or MCI alone (11, 15, 20–22, 28, 53–55, 78). In addition, it is critical to explore correlations with non-AD biomarkers, as patients with MBI can also progress to dementia with Lewy bodies and FTD (19, 23, 84, 85). Despite these limitations, these findings are promising and support our support for MBI as a new marker for preclinical diseases.

In summary, MBI is a validated neurobehavioral syndrome that reflects the neurobehavioral axis of the pre-dementia risk state and complements the neurocognitive axis represented by SCD and MCI. In this review, based on evidence from clinical cognitive assessments, neuroimaging, and neuropathology, we consider MBI as a non-cognitive marker of neurodegenerative disease that can be used as an indicator of the preclinical stage of dementia and that the MBI-C is a useful tool for identifying MBI. MBI facilitates the early detection of AD dementia and helps to select individuals at risk for AD dementia for observation and clinical trials, with a view to providing a window for early intervention and slowing its progression.

## Author contributions

PJ and EY conceived of and planned the layout and content of this review. PJ, EY, JX and ZL contributed to the literature review and drafting of individual sections of this work. YZ, YW and WS revised this work critically and contributed to the diagrams of it. All authors reviewed and edited this manuscript in its entirety for intellectual content, provided final approval of the version to be submitted for review and publication, and agree to be accountable for all aspects of the work including ensuring that questions related to the accuracy or integrity of any part of the work are appropriately investigated and resolved.

## Funding

This work was supported by the National Natural Science Foundation of China (grant number 8177051246). The funder will contribute toward the cost of open access publication fees.

## Conflict of interest

The authors declare that the research was conducted in the absence of any commercial or financial relationships that could be construed as a potential conflict of interest.

## Publisher’s note

All claims expressed in this article are solely those of the authors and do not necessarily represent those of their affiliated organizations, or those of the publisher, the editors and the reviewers. Any product that may be evaluated in this article, or claim that may be made by its manufacturer, is not guaranteed or endorsed by the publisher.
